# Review on Microbially Influenced Concrete Corrosion

**DOI:** 10.3390/microorganisms11082076

**Published:** 2023-08-12

**Authors:** Dongsheng Wang, Fang Guan, Chao Feng, Krishnamurthy Mathivanan, Ruiyong Zhang, Wolfgang Sand

**Affiliations:** 1 Key Laboratory of Marine Environmental Corrosion and Bio-Fouling, Institute of Oceanology, Chinese Academy of Sciences, Qingdao 266071, China; dongsheng937@163.com (D.W.); guanfang@qdio.ac.cn (F.G.); kritamathi@qdio.ac.cn (K.M.); 2 School of Civil Engineering, Qingdao University of Technology, Qingdao 266033, China; fengchao@qut.edu.cn; 3 Guangxi Key Laboratory of Marine Environmental Science, Institute of Marine Corrosion Protection, Guangxi Academy of Sciences, Nanning 530007, China; 4 Aquatic Biotechnology, University of Duisburg-Essen, 45141 Essen, Germany; 5 Institute of Biosciences, Freiberg University of Mining and Technology, 09599 Freiberg, Germany

**Keywords:** microorganisms, concrete, mechanism, research methodology, protection

## Abstract

Microbially influenced concrete corrosion (MICC) causes substantial financial losses to modern societies. Concrete corrosion with various environmental factors has been studied extensively over several decades. With the enhancement of public awareness on the environmental and economic impacts of microbial corrosion, MICC draws increasingly public attention. In this review, the roles of various microbial communities on MICC and corresponding protective measures against MICC are described. Also, the current status and research methodology of MICC are discussed. Thus, this review aims at providing insight into MICC and its mechanisms as well as the development of protection possibilities.

## 1. Introduction

**Figure 1:**
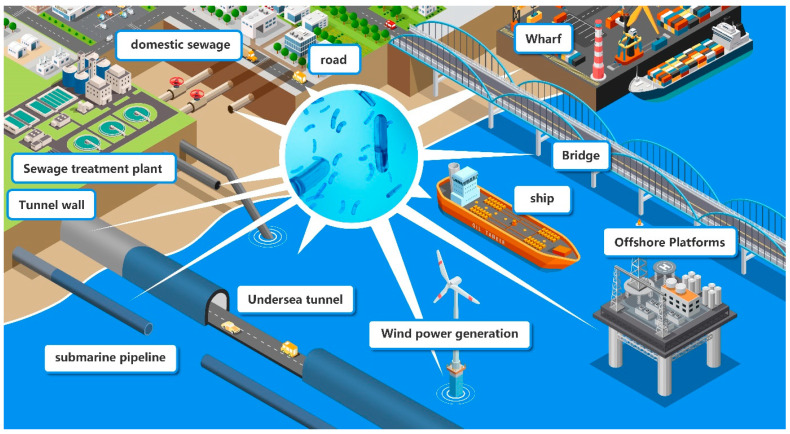
Overview on possible sites of MICC of urban infrastructure built with concrete.

The use of concrete has a profound impact on the development of a modern society [1]. Concrete is widely used in various industries and its use is increasing due to the development of urban infrastructure and marine engineering [2]. Concrete is a superior building material that is widely employed in a variety of urban infrastructure projects, including sewer pipes, offshore platforms and coastal infrastructure [3]. Among them, concrete used in sewage systems or offshore platforms is exposed to complex environments, which are prone to furthering the growth of microorganisms and subject to destruction with microbial activities (Figure 1).

Chemical, physical, mechanical and biological corrosions are the common types of concrete deterioration [4]. Among them, microbial corrosion is one of the most significant causes of the deterioration of concrete sewers [5]. According to reports, most of the sewer systems in Austria have been degraded significantly with microbial corrosion after 9 years of function [6]. It cost USD 400 million to replace 11% of a concrete pipe in Los Angeles, California, which had been damaged with microbial corrosion [7]. Estimations for sewage restoration in the US may range in costs up to USD 1.6 trillion in total [8]. It has been reported that a serious pipeline collapse occurs every 4 days in Beijing [9]. Obviously, concrete constructions are prone to microbial corrosion, causing significant losses and security disasters.

Microbially influenced concrete corrosion (MICC) [6,10,11] is a complex process involving microbiology, chemistry, environmental science, materials, civil engineering, etc. [12]. Due to the ubiquitous abundance of microorganisms and inevitable MICC problems, many countries need to allocate huge funds to repair and rebuild sewer systems. MICC has also become a prominent issue in concrete construction. Thus, a study on MICC aids in understanding its mechanisms for developing appropriate possibilities for countermeasures. The goal of this review is to evaluate the research on MICC and the prevention of concrete corrosion including protection measures. Also, this review intends to draw increased attention to this interdisciplinary subject to experts in microbiology, material science and civil engineering.

## 2. Concrete

Concrete has become an ideal building material because of its high strength, low cost, excellent processability and durability [13]. Over the last 200 years, the rapid development of urbanization has been closely related to the widespread use of concrete [14]. Concrete is a cementitious material made by mixing cement, sand, stone, water and the required admixtures in certain proportions, followed by compacting and curing under defined conditions [15]. Sand and stones serve as the aggregate and skeleton, respectively, in concrete constructions [16]. Cement and water combine to make mud, which wraps the aggregate and fills the gaps between the aggregate particles in concrete constructions [17]. Cementitious material has good workability before curing and a high strength after curing [18]. Further on, workability, strength, deformation and durability are properties that determine the quality of concrete [19,20,21]. Workability refers to the ability to facilitate various certain construction conditions to obtain uniform and dense concrete [22]. Strength includes the compressive, tensile, flexural, bending, shear and gripping strength of concrete [23]. Also, different buildings require varying levels of concrete strength, which are chosen based on the actual service scenario [24]. The deformation of concrete includes creep and shrinkage [25]. The creep of concrete is the continuous growth of strain over time under long-term stress. The shrinkage of concrete refers to the reduction in the volume of concrete. The shrinkage types include plastic shrinkage, chemical shrinkage, drying shrinkage and carbonation shrinkage [26]. Both the creep and shrinkage of concrete can cause cracking and reduce the strength of concrete [27]. The durability of concrete refers to the ability of concrete structures to sustain their safety and normal use under various environmental effects for a specified service life without additional costly reinforcements [28]. Concrete properties, structural design, construction and maintenance management are some factors influencing the durability of concrete [29]. Also, the durability of concrete is threatened with various processes caused by chemical, physical or microbial influences [27].

## 3. Research Status of Concrete Corrosion

Concrete can be destroyed with various environmental factors. This affects not only the aesthetics of a concrete structure but also shortens its durability. Concrete corrosion can be sub-divided into physical, chemical and biological [30]. Physical corrosion refers to the dissolution, expansion or shrinkage of certain concrete components as an influence of environmental factors such as heat, radiation, frost, etc. [31]. Physical influences, such as wet–dry cycles, wear failure and freeze–thaw cycles, damage the concrete structure and reduce its strength [32,33]. Among the physical factors, freeze–thaw cycles have the greatest impact on concrete strength [34].

**Figure 2:**
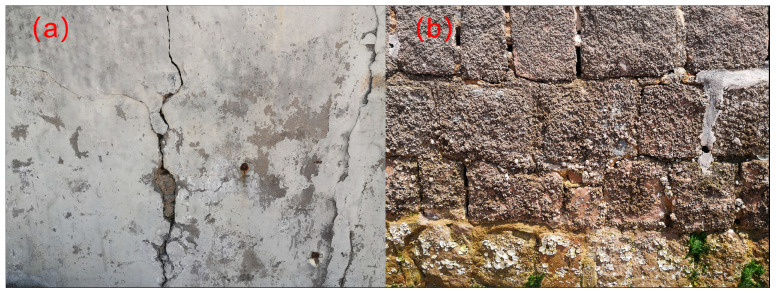
Selected photos of cracked concrete in a coastal environment (**a**), concrete with biological attachment (**b**); photographed in Qingdao, Shandong Province, China, 2023.

Chemical corrosion means the breakdown of concrete caused by a chemical reaction with, e.g., acids, alkalis and salts [35]. It results in a loss of binding material, strength reduction [36] and other problems [37]. Sulfate corrosion, an alkali aggregate reaction, carbonatization and chloride ion erosion (in the case of reinforced concrete) are the main types [38,39,40]. For example, sulfate enters the interior of the concrete and reacts with the calcium ion to produce the expansion product gypsum, and the continuous accumulation of gypsum will cause the cracking and destruction of the concrete [41]. Carbon dioxide infiltrates porous concrete and becomes dissolved in capillary liquid to form carbonic acid [42]. Carbonic acid reacts with calcium hydroxide, thereby reducing the alkalinity of concrete [42]. Figure 2a shows the cracking of concrete after chemical and physical corrosion in the coastal environment of Qingdao, China. Besides physical and chemical corrosion, (micro)organisms, such as bacteria, fungi and algae, can cause concrete damage by secreting metabolites [4,43,44]. Figure 2b shows concrete with biological attachment. Microbial influence on the corrosion of inorganic materials was summarized by Sand [45] into nine main categories; among them, mineral acids’ attacks, organic acids’ attacks and damage caused by salt stress are suitable for MICC. Currently, MICC is drawing increasing attention. In particular, concrete constructions like urban sewer systems or those in coastal areas have suffered greatly from severe microbial corrosion.

## 4. MICC

### 4.1. Microbial Communities in Sewage Environments

**Figure 3:**
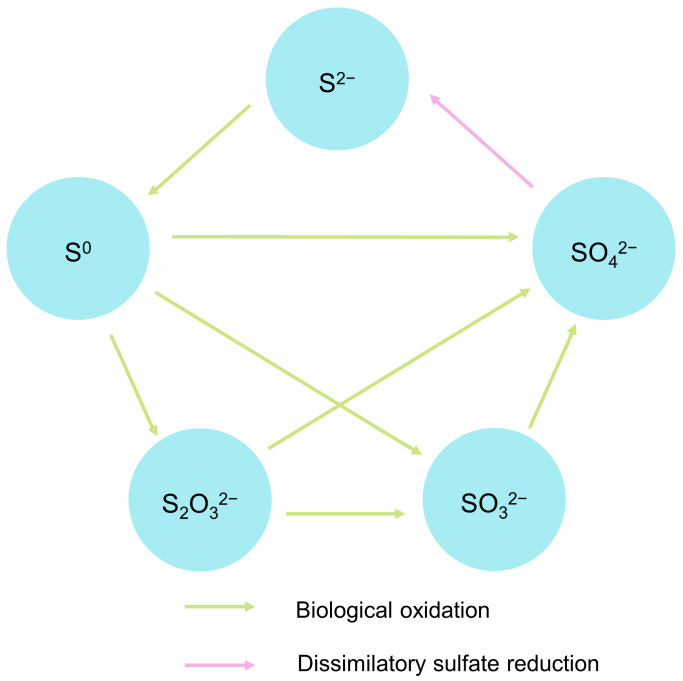
The sulfur cycle driven by microorganisms [53].

The composition of microbial communities involved in concrete corrosion needs to be studied to find effective ways of understanding the processes in MICC [46]. The metabolic activities of microorganisms are highly dependent on environmental conditions [47]. Changes in these conditions can have a significant effect on microbial community composition and abundance [48]. pH is one of the most important environmental factors for MICC. The pH of a concrete surface is alkaline. It changes due to carbonatization and environmental influences. In addition, microbial metabolites can cause pH decreases of the concrete surface, which cause damage to concrete [9]. pH changes strongly influence microbial abundance and dominant species, many of which are associated with sulfur and nitrogen metabolism [49]. Almost all microbes, such as bacteria, archaea, fungi and algae, are involved in MICC [50]. MICC is closely related to the sulfur cycle driven by microorganisms (Figure 3), especially in a sewer environment. Sulfate-reducing prokaryotes (SRP) and sulfur-oxidizing bacteria (SOB) that drive the sulfur cycle will be described in detail [51,52,53]. Hydrogen sulfide is emitted into sewers’ atmosphere due to sewage acidification and turbulence; then, it may react with oxygen into elemental sulfur [51,53]. Hydrogen sulfide and elemental sulfur can be converted to sulfuric acid with the metabolism of SOB [51,53]. The end product sulfate may be used again by SRP. Thus, the sulfur cycle is closed in a sewer environment [51,53].

Sand and Bock [54] found that *Acidithiobacillus (A.) thiooxidans* dominated the flora in heavily corroded concrete (pH about 1); *T. intermedia* (*T. intermedius*) and *T. novellus* dominated the flora in corroded concrete with a moderate pH (range of 3 to 6), and *A. thiooxidans* was not detectable if the pH of concrete was above 6. Zhou et al. [48] found that the bacterial abundance in freshwater MICC was significantly higher than in seawater MICC. Members of the genus Bacteroides were predominantly in freshwater, while Proteobacteria were predominantly in seawater. In addition, Proteobacteria, Synergistetes, Firmicutes and Thermotogae as well as SRP belonging to the class Deltaproteobacteria were found on concrete immersed in both freshwater and seawater [48]. Jiang et al. [55] simulated a sewer system, where wastewater was periodically inoculated onto surfaces of concrete samples. They found that SOB accounted for 80–90% of the total microbial community, with *A. thiooxidans* accounting for 35–50%. Grengg et al. [56] analyzed the microbial communities from heavily corroded concrete in an Austrian sewer system and found that *A. thiooxidans* and *A. ferrooxidans* were the dominant members. The pH of a concrete surface in a gravity sewer system changed from 10.5 to 3.1 after 20 days of exposure. *A. ferrooxidans*, *A. thiooxidans* and *A. caldus* were found to be the dominant species among SOB. They were closely associated with the biogenic sulfuric acid production [57]. Furthermore, the composition of bacterial communities is often related to the concrete type and their growth and metabolic activities are dependent on the surface pH [58,59]. Selected bacteria involved in MICC and their effects on concrete corrosion are shown in Table 1.

### 4.2. Sulfate-Reducing Prokaryotes

**Figure 4:**
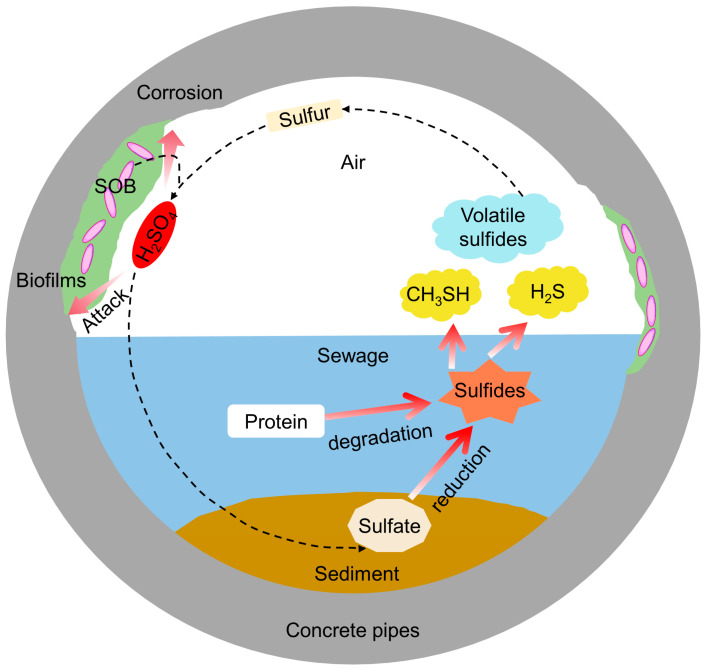
Schematic illustration of the main events involved in MICC of concrete exposed to sewer environments, modified from [53] with permission.

All living organisms excrete carbon dioxide as the end product of metabolism [47]. Carbon dioxide carbonizes concrete, which decreases the pH of the concrete surface [45]. Also, the pH of the concrete surface can be reduced with hydrogen sulfide gas. This pH reduction allows the adhesion of SOB and is thereby accelerating the corrosion of concrete [76]. H_2_S is produced with SRP metabolism. H_2_S is oxidized into S^0^ with chemical and biological action. H_2_S and S^0^ do not corrode concrete. So SRP does not corrode concrete, at least not directly, but produces H_2_S, which after oxidation with SOB/SOP, causes corrosion in the form of H_2_SO_4_ [54,77]. The main events involved in the MICC of concrete exposed to sewer environments are illustrated schematically in Figure 4.

### 4.3. Sulfur-Oxidizing Bacteria

**Figure 5:**
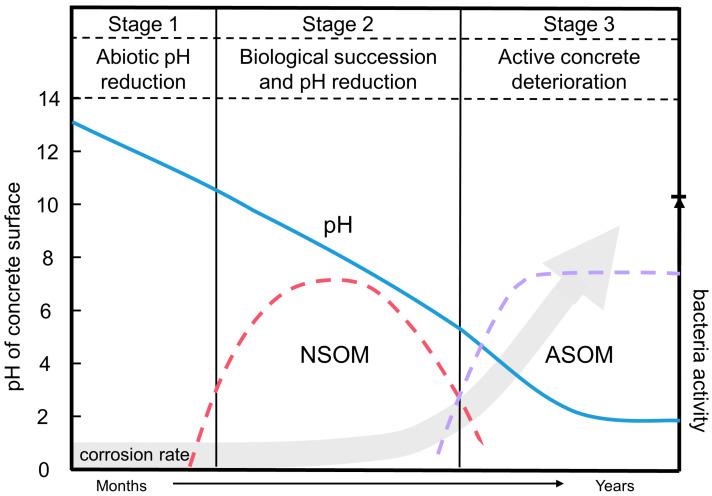
A three-stage model for the corrosion of concrete exposed to sewer environments, adapted from [58,79] with permission.

The pH of the concrete surface is significant for concrete degradation, because it influences the colonization, metabolism, growth and reproduction of microorganisms on concrete [54]. Sewage systems contain a complex community of microorganisms. Sulfur-oxidizing bacteria, such as neutrophilic sulfur-oxidizing microorganisms (NSOM) and acidophilic sulfur-oxidizing microorganisms (ASOM) have been found in concrete systems [54,78]. The abundances of NSOM and ASOM on concrete vary greatly depending on the sewer environment [79]. During the first few years of service, the concrete surface is strongly alkaline, with a pH of 11~13 [80], which hinders microbial colonization [81]. However, the pH of the concrete surface gradually decreases due to a continuous chemical attack with microbial metabolites in sewage such as carbon dioxide and hydrogen sulfide. The pH favorable for bacterial growth is attained if it is reduced to 9. Coupled with the larger humidity, suitable temperature and abundant nutrients in the sewer system, the NSOM begin to grow and reduce the pH of the concrete surface through their metabolism [58]. *Thiobacillus thioparus*, *T. novellus*, *T. neapolitanus* and *T. intermedia* are neutrophilic sulfur-oxidizing microorganisms, which are commonly and closely associated with concrete corrosion [69,77,79]. Under the influence of NSOM, the pH of the concrete surface can be reduced to 4–5. The low pH environment meets the growth requirements of ASOM. The growth of NSOM and ASOM as a function of pH, as well as the quality change of concrete at different stages, is shown in Figure 5.

SOB generate energy by oxidizing reduced inorganic sulfur compounds, e.g., sulfur (Equation (1)) and thiosulfate (Equation (2)) [82]. Biogenic sulfuric acid, which is the final product of microbial metabolism with SOB [82], reacts further with calcium carbonate, a carbonization product on the concrete surface, to produce gypsum (Equation (3)) [83]. The biogenic sulfuric acid may also enter the concrete through cracks and react with calcium hydroxide (Equation (4)) and calcium metasilicate (Equation (5)) to produce gypsum [60,83,84]. The latter will further react with silicate alumina (Equation (6)), causing swelling, cracking and eventually the deterioration of the concrete [60,83,84,85].

S + H_2_O + 1.5O_2_ → SO_4_^2−^ + 2H^+^ (1)

S_2_O_3_^2−^ + H_2_O + 2O_2_ → 2SO_4_^2−^ + 2H^+^ (2)

H_2_SO_4_ + CaCO_3_ → CaSO_4_ + H_2_CO_3_ (3)

H_2_SO_4_ + Ca(OH)_2_ → CaSO_4_ + 2H_2_O (4)

H_2_SO_4_ + CaO·SiO_2_·2H_2_O → CaSO_4_ + Si(OH)_4_ + H_2_O (5)

3CaSO_4_ + 3CaO·Al_2_O_3_·6H_2_O + 26H_2_O → 3CaO·Al_2_O_3_·3CaSO_4_·32H_2_O (6)

Sand et al. [54] established the relationship between the biodegradation of concrete and the number of *A. thiooxidans* (formerly *T. thiooxidans*). If the cell counts of the *A. thiooxidans* logarithm are below 6.8 ± 0.6 cell/cm^2^, the concrete corrosion is negligible; if the cell counts of the *A. thiooxidans* logarithm are 7.1 ± 0.7 cell/cm^2^, the concrete corrosion is medium; if the cell counts of the *A. thiooxidans* logarithm are 7.7 ± 0.5 cell/cm^2^, strong concrete corrosion is resulting. Milde et al. [69] detected thiobacilli from concrete with different degrees of corrosion in the Hamburg sewer system. They found a significant enrichment of thiobacilli on concrete surfaces above the sewage level. Satoh et al. [10] described that only one clone from the bottom biofilm sample belonged to SRP, while twelve clones from the intermediate biofilm and six clones from the eroded material belonged to SOB. Jiang et al. [55] found that SOB accounted for 80–90% of the total microbial community, with *A. thiooxidans* accounting for 35–50%. Grengg et al. [43] isolated and identified the bacterial species of heavily corroded concrete and found *A. ferrooxidans* to be prevalent. Okabe et al. [86] found that at least six SOB species were involved in concrete corrosion, with *Thiothrix* sp., *Thiobacillus plumbophilus*, *Thiomonas intermedia*, *Halothiol neapolitanus* (formerly *Halothiobacillus neapolitanus*), *Acidiphilium acidophilum* and *A. thiooxidans* being the most abundant. Also, *A. thiooxidans* accounted for 70% after a year of service. Gutiérrez-Padilla et al. [87] assessed the corrosion rate of concrete with a mixed culture of an NSOM strain (*Halothiol neopolitanus* ATCC 23641) and an ASOM strain (*A. thiooxidans* ATCC 8085). It amounted to 0.08 mm/year. In addition, the addition of NSOM in a mixed culture favored a reduction in pH from 5.3 to 3, thus allowing ASOM to grow. In the MICC field, ASOM are the dominant species on concrete surfaces if the pH has dropped below 4. The main ASOM associated with sulfuric acid production are *A. ferrooxidans*, *A. thiooxidans* and *A. caldus* [58,77].

### 4.4. Nitrifying Bacteria

Nitrifying bacteria oxidize ammonia and nitrite for energy generation and produce nitric acid. The acid reacts with alkaline binding materials like concrete. The product is calcium nitrate, which is water-soluble. Rain washes nitrates off a wall, thus causing a loss of concrete [50]. Thiobacilli are responsible for a degradation of concrete below the ground, and nitrifying bacteria are responsible for a degradation of concrete above the ground [45]. Sand and Bock [50] demonstrated in simulation experiments that nitrifying bacteria deteriorate concrete and sandstone with nitric acid production. The corrosive activity was comparable to a biogenic sulfuric acid attack.

### 4.5. Fungi

Similar to bacteria, fungi can colonize concrete structures and cause concrete degradation in environments with sufficient nutrients, meaning energy and organic carbon sources as well as favorable temperature and humidity [49]. Gu et al. [88] observed, with a microscopic investigation, that organic acids secreted by fungi can cause damage to concrete. In addition, the effects of *Thiobacillus* spp. (bacteria) and *Fusarium* spp. (fungi) on concrete corrosion were compared. The author [88] described that the corrosion of concrete was more severe in the environments with fungi than in the environments with bacteria, which indicated that the mass loss of the concrete inoculated with bacteria and fungi for 147 days was 18% and 24%, respectively. Fungi caused a greater mass loss in concrete, which may be related to the amount of Ca^2+^ released [88]. The asexual reproduction of fungi is rapid and common [89]. Fungi can germinate from spores and the hyphae start to grow out. With septum formation, they gradually form a mycelium [90,91,92]. Since hyphae are small, they allow the fungus to grow in concrete cracks and fissures [79,84]. Fungi influence concrete corrosion in two different ways [93]. Type one refers to the deterioration of concrete with corrosion products produced via a reaction between concrete and organic acids secreted by a fungus [93]. Most organic acids can react with Ca^2+^ to form water-soluble salts, resulting in Ca^2+^ releasing and the deterioration of concrete. Also, the fungal type and environmental conditions could also influence the formation of organic acids. Reactions of acetic acid and CH (Ca(OH)_2_), C-S-H (calcium and silicate hydrate) and ettringite (3CaO·Al_2_O_3_·3CaSO_4_·32H_2_O) in concrete are shown in Equations (7)–(9) [85,93], respectively. The second type means that the mycelium continues to develop in concrete fractures, putting mechanical force on the cracks as it grows. It further expands the extent of the crack and causes damage to the concrete structure [48,49,94,95,96,97]. The diameter of hyphae ranges from 2 to 6 μm [98]. Cracks in concrete range from a few microns to even a few millimeters [99,100]. It is feasible for the pores in concrete to contain water, which provides the possibility for the growth of fungi [93].

Ca(OH)_2_ + 2HC_2_H_3_O_2_ → Ca(C_2_H_3_O_2_)_2_ + H_2_O (7)

1.8C-S-H + 3.6HC_2_H_3_O_2_ → 1.8Ca(C_2_H_3_O_2_)_2_ + H_4_SiO_4_ + 1.6H_2_O (8)

Ettringite + 6HC_2_H_3_O_2_ → 3Ca(C_2_H_3_O_2_)_2_ + 2Al(OH)_3_ + 3CaSO_4_+ 26H_2_O (9)

**Figure 6:**
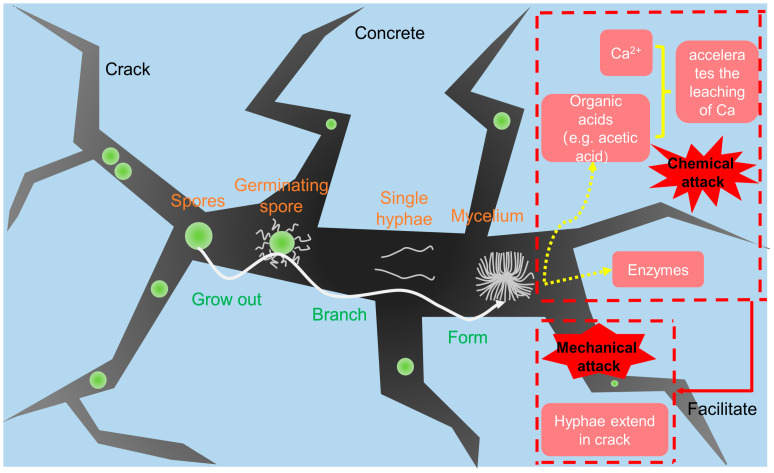
Schematic illustration of fungally influenced concrete corrosion [93].

Figure 6 depicts the colonization of concrete with fungi as well as the two types of fungally influenced concrete corrosion. The fungal metabolism alters the surrounding environment (e.g., pH), which is also influencing bacterial growth. These fungal–bacterial interactions influence the deterioration of materials [101]. Bhattacharyy et al. [102] evaluated the effects of three fungi on MICC including *Aspergillus tamarii*, *Aspergillus niger* and *Fusarium* sp. They found that *Aspergillus tamarii* caused the most severe mass loss, followed by *Aspergillus niger* and *Fusarium* sp. Chaudhuri et al. [103] found that the mass loss of concrete was 7.2% after 90 days of inoculation with *Aspergillus tamarii*, while the mass loss without fungi was 1.0%. Additionally, *Aspergillus tamarii* can also colonize concrete within a short period, cause cracks and produce calcium oxalate, thus accelerating calcium ion leaching and ultimately decreasing the strength of the concrete. George et al. [98] found that *Fusarium* sp. Could be used as a black biofilm to colonize the surface of concrete, which results in a pH reduction from 12 to 8 and a mass loss of 6.2 g after 1 year.

### 4.6. Factors Influencing MICC

Factors that influence microbial corrosion in sewer systems are mainly temperature, humidity, pH, oxygen content, water velocity and the residence time of sewage. These factors jointly interact with concrete [8,43]. MICC is usually inhibited with a high pH. Increased temperatures can increase corrosion by enhancing SRP to produce hydrogen sulfide more rapidly [104]. An oxygen-rich environment limits the biological activity of SRP and, thus, reduces hydrogen sulfide production [105]. SOB oxidize hydrogen sulfide and sulfur into sulfuric acid for metabolic energy. Hydrogen sulfide is the source of sulfur; the flora consists mainly of *A. thiooxidans* and the concrete is corroded severely. If thiosulfate is the source of sulfuric acid, the flora consists mainly of *Halothiol neapolitanus* and *Thiomonas intermedia*. In this situation, the concrete suffers from medium corrosion. Methylmercaptan, as an organic sulfur compound, results in negligible corrosion [71].

MICC is affected generally by concrete composition and preparation. For instance, high-strength concrete should have high compression resistance against crack formation. These parameters can limit the entry of biological sulfuric acid into concrete, thereby reducing its destruction [106]. Mori et al. [107] found that the most severe corrosion of concrete occurred around the sewage level and it decreased with increasing distance from the water level. The presence of nutrients, water and oxygen determined the maximal corrosion rate. The service life of concrete sewer pipes around the sewage water level was approximately 20 years with corrosion rates between 4.3 and 4.7 mm/year in their experience. The corrosion products varied depending on the pH. In particular, gypsum was the main corrosion product at a pH below 3, whereas ettringite was the predominant corrosion product at a pH above 3. Gutierrez et al. [108] found a significant relationship between SRP activity and pH. A 30% and 50% decrease in SRP activity occurred between pH 8.6 and 9.0, respectively, as compared to pH 7.6. Joesph et al. [109] found that carbon dioxide had a small effect on the reduction in concrete surface pH, if compared to hydrogen sulfide gas.

At present, research on MICC mainly focuses on in situ field studies and laboratory simulation tests [54,70,110]. Despite remarkable research, there is no unifying measure to combine laboratory experiments with engineering applications. In addition, the MICC mechanism needs to be studied further and a complete theory of the microbial corrosion of concrete needs to be established.

## 5. Test Methods

**Figure 7:**
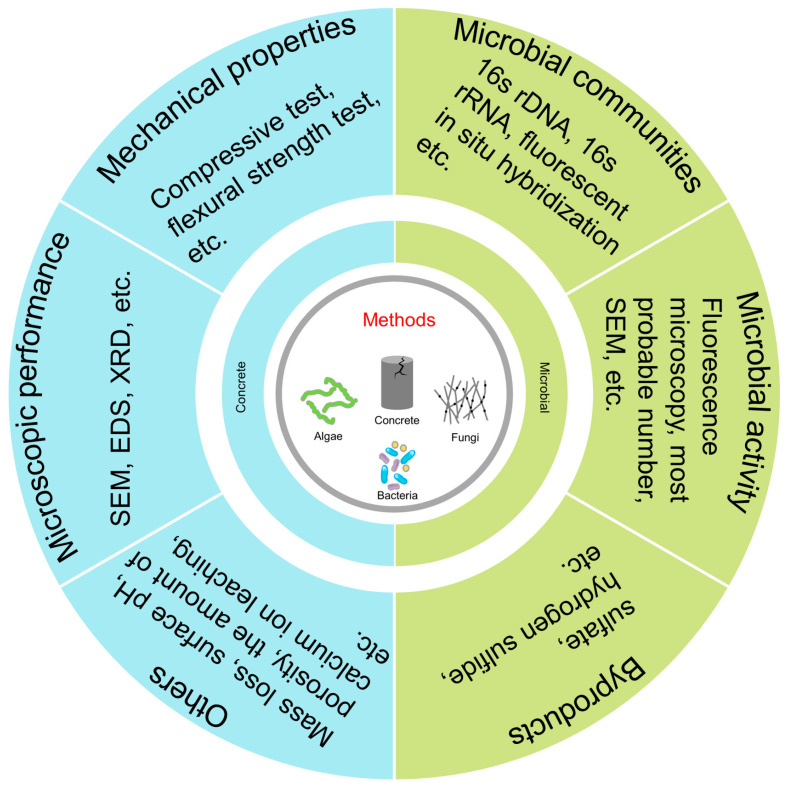
Overview of the methods for MICC. SEM: scanning electron microscopy; EDS: Energy Dispersive Spectrometer; XRD: X-ray diffraction.

The main test methods for the characterization of concrete are also usable to quantify deterioration with microorganisms. An overview of the research methods for MICC are shown in Figure 7.

### 5.1. Characterization of Concrete

Compressive tests, mass loss tests and an analysis of surface pH and porosity besides microscopic observation are common methods for assessing the quality of concrete [57,110,111,112]. They are also usable to quantify corrosion.

Wells and Melchers [113] measured the pH of a concrete surface at six different sites of a sewer system in Australia using a flat-faced pH probe (Extech pH 100). They found a surface pH of 10.1 at the beginning of the experiment and of 2.6~3.6 after 50 months of exposure. Their study revealed that the thickness of the corrosive layer of concrete increased in a nonlinear manner with time. Concrete permeability is a physical property that influences the rate of penetration of water, gas and other substances into concrete and also microbial cells. It is determined with the size, number, distribution and connectivity of pores inside the concrete [114]. The permeability and porosity of concrete are directly proportional [115]. The changes in concrete porosity caused with the penetration of corrosion media (e.g., SO_4_^2−^, Cl^−^ and H^+^) can lead to structural changes in concrete. Kong et al. [57] found that permeability is an important parameter, which characterizes the deterioration of a concrete structure. Wang et al. [116] found that the corrosion product gypsum adhered to the surface of concrete coupons when exposed to a mixed freshwater effluent. An NSOM biofilm adhered to a concrete surface can penetrate concrete through cracks and pores.

Huber et al. [117] characterized concrete deterioration by measuring the concrete surface pH and mass loss and analyzing elemental concentrations (carbon, calcium, silicon, phosphorus and sulfur) using laser ablation inductively coupled plasma mass spectrometry. Calcium ion leaching is an indicator of structural damage of concrete. Therefore, changes in dissolved calcium concentration reflect the extent of structural damage. The elemental distribution is used to characterize the structural and compositional changes in concrete after a chemical attack with sulfuric acid.

Grengg et al. [56] investigated concrete corrosion with quantitative elemental distribution images of aluminum, calcium, iron, magnesium, silicon and sulfur using an electron probe microanalysis analysis. Although measuring free calcium does not provide the amount of total calcium leached from the concrete, the relative concentration of calcium leached may be used to characterize the degree of concrete corrosion [118]. The morphology of corroded concrete is generally observed with a Scanning Electron Microscope (SEM) and the composition of corrosion material is analyzed with EDS and X-ray fluorescence. In addition to a physical, chemical and biological analysis, some researchers have also used modeling to measure the degree of concrete deterioration [113,116,119]. Li et al. [120] evaluated three data-driven models for the prediction of the life span of sewer systems based on the estimates of the corrosion initiation time and corrosion rate. They found that an artificial neural network and adaptive neurological fuzzy inference system models performed better than multiple linear regression models for corrosion prediction. Wells and Melchers [100] proposed a first-pass model to predict the rate of sewer concrete corrosion as a function of time and of the local sewer gas temperature, the relative humidity and the H_2_S concentration. Comparing the model predictions against reported corrosion rates revealed a good agreement. There are several methods for measuring the degree of concrete deterioration; however, there is no uniform standard for indicating the degree of deterioration worldwide. Therefore, a consistent and comprehensive system for characterizing MICC is needed.

### 5.2. Detection of Microorganisms and Characterization of Microbial Activity

There are several methods for characterizing microbial activity in MICC. Signature lipid biomarker fatty acids of polar lipids (PLFA) can be utilized to define the biomass and community structure of microbial consortia in biofilms, soils and sediments [108]. Kerger et al. [70] indicated that PLFA patterns are sufficiently specific to define the presence of acid-producing thiobacilli. This facilitates the identification of bacterial communities in corroded concrete pipes. In situ tests on sewer concrete revealed a dark grey gel-like biofilm with a thickness of 3 mm on the surface of a mortar submerged in an effluent [10]. Also, microsensor testing showed that the biofilm thickness on the mortar specimens was around 1000 μm after 7 days of installation, reaching 1500 μm after 28 days and becoming even thicker after 105 days [10]. A microsensor can be used to measure pH, hydrogen sulfide and oxygen concentration at different thicknesses within a biofilm. All parameters have a significant impact on the in-depth investigation of microbial concrete corrosion [10]. In general, bacterial concentrations are obtained by measuring biomass mass [121,122]. Sand and Bock [123] put concrete cubes into flasks with a mineral salt solution and incubated them on a rotary shaker for 90 min for the detachment of loose material and of adhering microorganisms. The resulting suspension was used to assess the concentration of bacteria on concrete surfaces. Kong et al. [124] analyzed the porosity of biofilms by calculating the ratio of the void area to the total area using a laser scanning confocal microscope. The biofilm and surface porosity are closely related to the effluent concentration, biofilm growth time and density of the biofilm. Khan et al. [125] observed the surface of concrete after 12 months of exposure to a natural aggressive sewer environment using SEM and found microorganisms appeared as black nodules in the microstructure, usually less than 10 μm in size.

The presence and distribution of bacteria can be observed with fluorescence microscopy after live/dead cell staining. SYTO9 and propidium iodide are common chemicals to stain live and dead cells, respectively [56]. Jiang et al. [55] characterized the activity of SOB with the sulfide uptake rate and found that the activity of SOB and corrosion rate of concrete were highest at a concentration of hydrogen sulfide of 25 ppm. Gutiérrez-Padilla et al. [87] found that the pH of the corrosive system decreased from 6.8 to 3.4 over 40 days with SOM, including *Halothiol neopolitanus* ATCC 641, *T. thioparus* ATCC 23646, *A. thiooxidans* ATCC 8085 and *A. cryptum* ATCC 33463. Those results showed that the decrease in pH was associated with biogenic sulfuric acid produced with SOB. One of the main byproducts of SOB activity is sulfate, whose concentration increased to 939 mg/L within 10 days, reaching a maximum of 1.85 g/L in their tests [87]. Both the decrease in pH and the increase in sulfate were related to the activity of SOB, but the relationship between pH or sulfate and the number of SOB was not introduced [87]. Sand et al. [54] indicated that the degradation of concrete was related to the number of *A. thiooxidans* attached to the concrete surface, and established a relationship between them.

## 6. Protection against MICC

The protection of concrete against MICC is important because it causes considerable costs. Studying the mechanism of MICC can help in the development of targeted protection technologies. The protection technologies of concrete from MICC are focused mainly on three aspects. The first is to limit the chemical attack and damage of concrete caused by limiting microbial activity. Secondly, a modification of concrete shall increase the resistance to seepage and cracking, and provide antibacterial properties [111,126]. Third, concrete surface treatments, such as the application of an anti-corrosion coating, may effectively control microbial activity and prevent the contact of corrosive substances such as biogenic sulfuric acid with concrete. Concrete surface protection is an efficient strategy for protecting concrete against microbial corrosion [127,128].

### 6.1. Inactivation of Microorganisms

Microbial activity is one of the most important factors influencing MICC [47]. Therefore, inhibiting microbial activity is an effective way to control MICC. Wang et al. [110] suggested various strategies to protect concrete construction from SRP activities, such as increasing the dissolved oxygen level in sewer systems to reduce the anaerobic conditions by accelerating water flow or reducing the sulfur source for SRP by altering the redox conditions in sewer systems. Microbial activities can be limited by the use of various biocides such as halogenated compounds; quaternary ammonium compounds; heterocyclic amines; iodopropyl compounds; copper, zinc, lead and nickel metal oxides; copper, zinc, lead and manganese phthalocyanines; tungsten and tungsten compounds; silver; organotin; etc. [129,130]. These biocides can protect concrete from microorganisms by inhibiting their metabolism and reproduction, thus reducing biogenic sulfuric acid production. However, biocides not only affect microorganisms but also have uncertain effects on properties of concrete [131]. Kong et al. [132] found a considerable number of dead microorganisms on concrete that had been treated with copper phthalocyanine, and reported that this compound had an excellent bactericidal activity and increased the workability and strength of the concrete. Sodium bromide, zinc oxide and dodecyl dimethyl benzyl ammonium chloride inhibit microorganisms, but are harmful to concrete [132]. The workability of concrete and the compressive strength of concrete decrease as the amount of zinc oxide increases [132], which may be related to the absorption and drying properties of zinc oxide that prevent cement hydration [132]. The compressive strength of concrete with 0.1% dodecyl dimethyl benzyl ammonium chloride decreased by 25% at 28 days [132]. The chloride ion in dodecyl dimethyl benzyl ammonium chloride can cause Ca^2+^ leaching [132]. Etim et al. [133] found that SRP concentration and biofilm thickness on concrete surfaces were significantly reduced if an organic silicon quaternary ammonium salt was used as a biocide and improved the corrosion resistance of concrete. Okeniyi et al. [134] found that the addition of C_10_H_18_N_2_Na_2_O_10_ (ethylenediaminetetra-acetic acid disodium salt) to concrete improved concrete corrosion resistance in a simulated marine environment. Voicu et al. [135] compared the effects of silica and zinc oxide nanoparticles on concrete properties and found that the addition of silica nanoparticles improved the concrete’s mechanical strength, abrasion resistance, durability and high temperature resistance, but failed in improving the antimicrobial properties of concrete. In addition, concrete with the addition of ZnO nanoparticles inhibited the growth of biofilms compared to control concrete. Yamanaka et al. [136] studied the inhibitory effects of calcium formate, sodium formate and ammonium formate on the growth of *Halothiol neapolitanus* ATCC 23638, *A. thiooxidans* IFO 13724 and *A. ferrooxidans* JCM 7811 if these salts were mixed with cement. They found that calcium formate completely inhibited the growth of all tested strains.

Sugio et al. [137] reported that the growth inhibition of the Fe-oxidizing bacterium *A. ferroxidans* with sodium tungstate was concentration-dependent. Ferrous iron can be oxidized with *A. ferroxidans* [137]. The relationship between the concentration of sodium tungstate and the growth inhibition of *A. ferroxidans* was summarized from two aspects, the cell number and ferrous iron concentration (Table 2) [137]. Increased concentrations (0.05 mmol/L) of sodium tungstate strongly inhibited the growth in contrast to a low concentration (0.2 mmol/L). Nowadays, different types of biocides are used in a variety of ways. For instance, they can be incorporated into the internal structure of concrete as fillers [138], applied to the surface of concrete as coatings [139] or added directly to corrosive environments [140]. However, few studies have been conducted on the effects of biocides on concrete properties, and some of them have shown that biocides have negative effects on concrete properties [141]. The toxicity of biocides is another serious problem, which can pose risks to wastewater treatment plants and the environment [142].

### 6.2. Concrete Modification

Improving the corrosion resistance of concrete through modification can be another way to control MICC. The modification of concrete can be achieved by changing the structure of concrete. The permeation resistance of concrete is influenced significantly with biological corrosion and is closely linked to the pore structure of concrete. Due to its low porosity and small pore size, aerated concrete has a significantly better permeation resistance and a better acid corrosion resistance than ordinary concrete [106]. Another effective method for concrete modification is the addition of functional materials to improve the properties of concrete [143]. Song et al. [144] found that concrete containing 1% or 2% anaerobic granular sludge had 15% and 55% reduced corrosion rates, respectively, compared to control concrete without anaerobic granular sludge. The addition of anaerobic granular sludge increased the pH of the concrete surface and, thus, the total relative abundance of corrosion-causing microorganisms was reduced. That bio-concrete can significantly reduce the production of biogenic sulfuric acid with microorganisms, thus reducing the corrosion of concrete [144].

The emergence of nanomaterials is a major advancement in material sciences. The addition of nanomaterials to concrete has been shown to significantly improve the performance of concrete by inhibiting the metabolism of microorganisms [145,146]. Klapiszewska et al. [147] investigated concrete performance with the addition of ZnO/lignin and ZnO-SiO_2_/lignin as admixtures and found that concrete containing ZnO/lignin had excellent antimicrobial properties and that ZnO determined the antimicrobial properties of this concrete. In the case of a ZnO-SiO_2_/lignin mixture, an addition of SiO_2_ reduced the ZnO content, which caused a decrease in antimicrobial properties.

Acid-resistant concrete can be prepared and improved by adding silica fume, kaolin and low-calcium fly ash to the concrete. This also improves the concrete resistance against microbial corrosion [148]. Usman and Sam [149] stated that incorporating metakaolin into concrete caused a superior resistance to a sulfuric acid attack compared to ordinary concrete. Polymers are also good fillers and concrete containing polymers such as styrene, acrylate, acrylic acid, butylbenzene, vinyl polymers and polyethylene terephthalate showed less corrosion compared to ordinary concrete [150,151].

Adding a biocide into concrete is one of the methods to produce antimicrobial concrete. Antimicrobial concrete containing zinc oxide and sodium bromide has been shown to have an inhibitory effect on microorganisms [136,139,152,153,154].

The modification of the concrete surface is another method. Hayek et al. [155] investigated microbial adhesion on concrete surfaces by reducing the roughness of concrete and found that the roughness influences the microbial adhesion, meaning a reduced roughness decreases microbial adhesion.

Fiber reinforcement can help to protect concrete. Biogenic sulfuric acid can cause the production of calcium aluminate, a swelling product in concrete, which causes cracking. A fiber reinforcement can improve the cracking resistance of concrete and thus reduce damage caused by swelling [156].

Currently, concrete modification can only reduce microbial corrosion to a certain extent and cannot completely eliminate microbial damage. Therefore, further efforts are needed to develop better methods for concrete modification to resist MICC.

### 6.3. Coatings

Protective concrete coatings are often classified into two types: inert coatings and antibacterial coatings [157,158]. The protective mechanism of inert coatings acts as a barrier to prevent biological sulfuric acid from coming into contact with concrete. The protective mechanism of antibacterial coatings aids in limiting or inactivating microbes and their activity, thereby reducing biological sulfuric acid production [9]. For highly corroded concrete sewer pipes, the sewer pipes can be protected with resins or glass-fiber-reinforced resins as inner wall coatings [50]. Kamarul Asri et al. [159] found that silver ions had an antibacterial effect when they studied silver-ion-modified zeolite–polyurethane coatings on mortar surfaces in an aqueous solution. Merachtsaki et al. [160] investigated the pH of concrete surfaces coated with calcined magnesium powder (MgO) and magnesium hydroxide powder [Mg(OH)_2_] in different proportions of [Mg(OH)_2_] (0%, 20%, 40% and 60%) and found that the pH of the concrete surface remained alkaline under the attack of biological sulfuric acid, confirming that these coatings protect concrete from a microbial attack. De Muynck et al. [153] found that the mass loss of concrete with an epoxy coating was 1.9 ± 0.4 g in microbial corrosion simulation environments. However, the mass loss of silver–copper-zeolite-modified concrete, antimicrobial-fiber-modified concrete and sewer pipe reference concrete was 9.4 ± 0.8 g, 9.1 ± 0.4 g and 18.7 ± 1.0 g, respectively [153]. The author concluded that the best protective performance was obtained with an epoxy coating. Haile et al. [161] stated that the pH of concrete pipes coated with copper oxide nanoparticles began to increase on the 5th day in their tests, owing to the inhibition of bacterial activity. Vaidya et al. [162] used electrodeposition to deposit copper on the concrete surface to study and overcome the problems of the poor bonding of a coating to concrete, besides shrinkage and spalling that occurred in the original spraying process. Roghanian et al. [163] modified a concrete surface antibacterial coating with a composite of zinc and bentonite as a carrier and found that the composite coating showed good bonding strength after corrosion with biogenic acids. The mortar samples coated with the composite coating showed a strength loss of 35% after corrosion, compared with the 73% strength loss of normal concrete.

Despite the widespread usage of coatings for concrete protection, certain drawbacks such as the bonding between a coating and the concrete and the impact of a coating on the performance of concrete warrant more research.

## 7. Perspectives and Concluding Remarks

The age of sewer systems worldwide is increasing. Thus, the problems caused by MICC are becoming more and more serious, and research on the mechanisms, influencing factors, corrosion models and protective measures for MICC is therefore becoming increasingly important. However, as MICC involves many disciplines (biology, material science, engineering structures, etc.), interdisciplinary research also poses numerous difficulties for researchers. There are fundamental and scientific problems that still exist with MICC. These subjects should attract more attention from researchers, in addition to the willingness to perform interdisciplinary work.

(1) Although remarkable achievements have been made for MICC, further research is needed due to the diversity of microbial species and differing environments. This review focuses on MICC processes in sewerage systems. It is worthwhile to also study and investigate MICC involved in a marine environment using established protocols, considering the increased demand for concrete in marine engineering.
(2) There is presently no established model for MICC prediction and no unified standard to incorporate the large number of data obtained in specific experimental conditions. Therefore, how to make the data obtained in a laboratory or the data obtained from field tests applicable to a wide range still awaits further efforts.
(3) The fundamental purpose of research on MICC is to protect concrete, but there are some drawbacks or undiscovered effects of various protective measures. For instance, biocides have an excellent performance in inhibiting the growth of microorganisms, but their impact on the performance of concrete and the environment may be problematic. Modifications of concrete and concrete coatings can protect concrete to a certain extent, but there are still many inevitable challenges in the actual engineering application, such as the cost, construction process and environmental impact issues.

## Figures and Tables

**Table 1:** Selected bacteria involved in concrete corrosion.

Bacteria	Effect	Species	References
SRP	Produce available H_2_S and S^0^ (polythionates)	*Desulfovibrio*	[60,61,62,63,64]
		*Desulfobacter*	[61,65,66]
		*Desulfobulbus*	[61,62,63,67]
		*Desulforegula*	[61,68]
SOB	SOB convert H_2_S and S^0^ (polythionates) to biogenic sulfuric acid. Biological sulfuric acid attacks concrete at the surface and, via cracks, reacts with calcium hydroxide to form gypsum, which causes cracking and deterioration of concrete	*Acidthiobacillus* *ferrooxidans*	[56,57]
		*Halothiobacillus*	[48]
		*Thiobacillus thioparus*	[9]
		*Thiomonas intermedia*	[69,70,71,72]
		*Acidthiobacillus* *ferrooxidans*	[73,74]
		*Acidthiobacillus* *thiooxidans*	[55,56,57,64,75]
		*Thiobacillus thiooxidans*	[69,70,71]
		*Thiobacillus novellus*	[71]

**Table 2:** The relationship between the concentration of sodium tungstate and the growth inhibition of *A. ferroxidans* [137].

Na_2_WO_4_ (mM)	Cell Growth (Cells/mL)	Concentration of Fe^2+^ (mM)
0	10^8^	0
0.01	10^7^	0
0.05	10^6^	20
0.1	10^5^	70
0.2	0	110

## Data Availability

No new data were created.

## References

[1] Behera M., Bhattacharyya S.K., Minocha A.K., Deoliya R., Maiti S. (2014). Recycled aggregate from C&D waste & its use in concrete—A breakthrough towards sustainability in construction sector: A review. Constr. Build. Mater..

[2] Somaiya P., Bhogayata A. (2023). A systematic conditional assessment of strength and durability damage of concrete structures in marine environments. Mater. Today Proc..

[3] Qu F., Li W., Dong W., Tam V.W.Y., Yu T. (2021). Durability deterioration of concrete under marine environment from material to structure: A critical review. J. Build. Eng..

[4] Umar M., Fathima N., Haji Sheik Mohammed M.S., Hemalatha S. (2019). Modified cement composites for protection against microbial induced concrete corrosion of marine structures. Biocatal. Agric. Biotechnol..

[5] Luimes R.A., Scheperboer I.C., Suiker A.S.J., Bosco E., Clemens F.H.L.R. (2022). Effect of biochemical attack on the mechanical performance of used concrete sewer pipes. Constr. Build. Mater..

[6] Grengg C., Mittermayr F., Baldermann A., Böttcher M.E., Leis A., Koraimann G., Grunert P., Dietzel M. (2015). Microbiologically induced concrete corrosion: A case study from a combined sewer network. Cem. Concr. Res..

[7] Gonzalez D., Keeling D., Thompson H., Larson A., Denby J., Curtis K., Yetka K., Rondini M., Yeargan E., Egerton T. (2020). Collection system investigation microbial source tracking (CSI-MST): Applying molecular markers to identify sewer infrastructure failures. J. Microbiol. Methods.

[8] Pagaling E., Yang K., Yan T. (2014). Pyrosequencing reveals correlations between extremely acidophilic bacterial communities with hydrogen sulphide concentrations, pH and inert polymer coatings at concrete sewer crown surfaces. J. Appl. Microbiol..

[9] Rong H., Zhang S., Ma G., Zheng X., Qian C., Zhang L., Zhang Y., Xu R. (2021). Formation, growth and corrosion effect of sulfur oxidizing bacteria biofilm on mortar. Constr. Build. Mater..

[10] Satoh H., Odagiri M., Ito T., Okabe S. (2009). Microbial community structures and in situ sulfate-reducing and sulfur-oxidizing activities in biofilms developed on mortar specimens in a corroded sewer system. Water Res..

[11] Ling A.L., Robertson C.E., Harris J.K., Frank D.N., Kotter C.V., Stevens M.J., Pace N.R., Hernandez M.T. (2015). High-Resolution Microbial Community Succession of Microbially Induced Concrete Corrosion in Working Sanitary Manholes. PLoS ONE.

[12] Lv J., Mao J., Ba H. (2015). Influence of marine microorganisms on the permeability and microstructure of mortar. Constr. Build. Mater..

[13] Maruyama I., Lura P. (2019). Properties of early-age concrete relevant to cracking in massive concrete. Cem. Concr. Res..

[14] Bagga M., Hamley-Bennett C., Alex A., Freeman B.L., Justo-Reinoso I., Mihai I.C., Gebhard S., Paine K., Jefferson A.D., Masoero E. (2022). Advancements in bacteria based self-healing concrete and the promise of modelling. Constr. Build. Mater..

[15] Zhao H., Zhang L., Wu Z., Liu A., Imran M. (2023). Aggregate effect on the mechanical and fracture behaviours of concrete. Int. J. Mech. Sci..

[16] Kumar L., Thanappan S., Mekonnen E., Mulugeta D., Chala G. (2021). Effect of fly ash and sand stone slurry on mechanical properties of concrete materials. Mater. Today Proc..

[17] Amini K., Vosoughi P., Ceylan H., Taylor P. (2019). Effect of mixture proportions on concrete performance. Constr. Build. Mater..

[18] Şimşek O., Pourghadri Sefidehkhan H., Gökçe H.S. (2022). Performance of fly ash-blended Portland cement concrete developed by using fine or coarse recycled concrete aggregate. Constr. Build. Mater..

[19] Papachristoforou M., Mitsopoulos V., Stefanidou M. (2018). Evaluation of workability parameters in 3D printing concrete. Procedia Struct. Integr..

[20] Gao P., Chen Y., Huang H., Qian Z., Schlangen E., Wei J., Yu Q. (2020). Investigation of drying-induced non-uniform deformation, stress, and micro-crack propagation in concrete. Cem. Concr. Compos..

[21] Yang L., An X., Du S. (2021). Estimating workability of concrete with different strength grades based on deep learning. Measurement.

[22] Olofinnade O., Ogara J. (2021). Workability, strength, and microstructure of high strength sustainable concrete incorporating recycled clay brick aggregate and calcined clay. Clean. Eng. Technol..

[23] Fantu T., Alemayehu G., Kebede G., Abebe Y., Selvaraj S.K., Paramasivam V. (2021). Experimental investigation of compressive strength for fly ash on high strength concrete C-55 grade. Mater. Today Proc..

[24] Meng L., Zhang C., Wei J., Li L., Liu J., Wang S., Ding Y. (2023). Mechanical properties and microstructure of ultra-high strength concrete with lightweight aggregate. Case Stud. Constr. Mater..

[25] Nastic M., Bentz E.C., Kwon O.-S., Papanikolaou V., Tcherner J. (2019). Shrinkage and creep strains of concrete exposed to low relative humidity and high temperature environments. Nucl. Eng. Des..

[26] Li L., Dao V., Lura P. (2021). Autogenous deformation and coefficient of thermal expansion of early-age concrete: Initial outcomes of a study using a newly-developed Temperature Stress Testing Machine. Cem. Concr. Compos..

[27] Amran M., Al-Fakih A., Chu S.H., Fediuk R., Haruna S., Azevedo A., Vatin N. (2021). Long-term durability properties of geopolymer concrete: An in-depth review. Case Stud. Constr. Mater..

[28] Douglas Hooton R. (2019). Future directions for design, specification, testing, and construction of durable concrete structures. Cem. Concr. Res..

[29] Zhang B., Zhu H. (2023). Durability of seawater coral aggregate concrete under seawater immersion and dry-wet cycles. J. Build. Eng..

[30] Panesar D.K., Zhang R., Narneni S.R. (2021). Chemical and physical approaches to improve the properties of concrete for application to nuclear related structures. Nucl. Eng. Des..

[31] Ibrahim M.A., Sharkawi A.E.-D.M., El-Attar M.M., Hodhod O.A. (2018). Assessing the corrosion performance for concrete mixtures made of blended cements. Constr. Build. Mater..

[32] Liu D., Tu Y., Sas G., Elfgren L. (2021). Freeze-thaw damage evaluation and model creation for concrete exposed to freeze–thaw cycles at early-age. Constr. Build. Mater..

[33] Gacu J.G., Sim A.A.M. (2022). Effect of marble microparticles as additive on the physical and mechanical properties of concrete mixes. Mater. Today Proc..

[34] Smith S.H., Qiao C., Suraneni P., Kurtis K.E., Weiss W.J. (2019). Service-life of concrete in freeze-thaw environments: Critical degree of saturation and calcium oxychloride formation. Cem. Concr. Res..

[35] Uthaman S., George R.P., Vishwakarma V., Harilal M., Philip J. (2019). Enhanced seawater corrosion resistance of reinforcement in nanophase modified fly ash concrete. Constr. Build. Mater..

[36] Çullu M., Arslan M. (2014). The effects of chemical attacks on physical and mechanical properties of concrete produced under cold weather conditions. Constr. Build. Mater..

[37] Kiliswa M.W., Scrivener K.L., Alexander M.G. (2019). The corrosion rate and microstructure of Portland cement and calcium aluminate cement-based concrete mixtures in outfall sewers: A comparative study. Cem. Concr. Res..

[38] Shevtsov D.S., Zartsyn I.D., Komarova E.S. (2021). Relation between resistivity of concrete and corrosion rate of reinforcing bars caused by galvanic cells in the presence of chloride. Cem. Concr. Compos..

[39] Pan J., Wang W., Wang J., Bai Y., Wang J. (2022). Influence of coarse aggregate size on deterioration of concrete affected by alkali-aggregate reaction. Constr. Build. Mater..

[40] Klein N., Gómez E.D., Duffó G.S., Farina S.B. (2022). Effect of sulphate on the corrosion of reinforcing steel in concrete. Constr. Build. Mater..

[41] Sun D., Huang C., Cao Z., Wu K., Zhang L. (2021). Reliability assessment of concrete under external sulfate attack. Case Stud. Constr. Mater..

[42] Zhang Y., Gu L., Zhang Q. (2022). Durability of manufactured sand concrete in atmospheric acidification environment. Case Stud. Constr. Mater..

[43] Grengg C., Mittermayr F., Ukrainczyk N., Koraimann G., Kienesberger S., Dietzel M. (2018). Advances in concrete materials for sewer systems affected by microbial induced concrete corrosion: A review. Water Res..

[44] Song Y., Tian Y., Li X., Wei J., Zhang H., Bond P.L., Yuan Z., Jiang G. (2019). Distinct microbially induced concrete corrosion at the tidal region of reinforced concrete sewers. Water Res..

[45] Sand W. (1997). Microbial mechanisms of deterioration of inorganic substrates—A general mechanistic overview. Int. Biodeterior. Biodegrad..

[46] Raza S., Kang K.H., Shin J., Shin S.G., Chun J., Cho H.U., Shin J., Kim Y.M. (2023). Variations in antibiotic resistance genes and microbial community in sludges passing through biological nutrient removal and anaerobic digestion processes in municipal wastewater treatment plants. Chemosphere.

[47] Xu D., Gu T., Lovley D.R. (2023). Microbially mediated metal corrosion. Nat. Rev. Microbiol..

[48] Zhou J., Yin S., Fu Q., Wang Q., Huang Q., Wang J. (2021). Microbial-induced concrete corrosion under high-salt conditions: Microbial community composition and environmental multivariate association analysis. Int. Biodeterior. Biodegrad..

[49] Maresca J.A., Moser P., Schumacher T. (2016). Analysis of bacterial communities in and on concrete. Mater. Struct..

[50] Sand W., Bock E. (1991). Biodeterioration of mineral materials by microorganisms—Biogenic sulfuric and nitric acid corrosion of concrete and natural stone. Geomicrobiol. J..

[51] Thierry D., Sand W., Marcus P. (2002). Microbially Influenced Corrosion. Corrosion Mechanisms in Theory and Practice.

[52] Liu H., Hu Z., Zhou M., Zhang H., Zhang X., Yue Y., Yao X., Wang J., Xi C., Zheng P. (2022). PM2.5 drives bacterial functions for carbon, nitrogen, and sulfur cycles in the atmosphere. Environ. Pollut..

[53] Li X., Kappler U., Jiang G., Bond P.L. (2017). The Ecology of Acidophilic Microorganisms in the Corroding Concrete Sewer Environment. Front. Microbiol..

[54] Sand W., Bock E. (1990). Biodeterioration of concrete by thiobacilli and nitrifying bacteria. Matér. Tech..

[55] Jiang G., Zhou M., Chiu T.H., Sun X., Keller J., Bond P.L. (2016). Wastewater-Enhanced Microbial Corrosion of Concrete Sewers. Environ. Sci. Technol..

[56] Grengg C., Mittermayr F., Koraimann G., Konrad F., Szabó M., Demeny A., Dietzel M. (2017). The decisive role of acidophilic bacteria in concrete sewer networks: A new model for fast progressing microbial concrete corrosion. Cem. Concr. Res..

[57] Li X., O’Moore L., Song Y., Bond P.L., Yuan Z., Wilkie S., Hanzic L., Jiang G. (2019). The rapid chemically induced corrosion of concrete sewers at high H_2_S concentration. Water Res..

[58] Islander R.L., Devinny J.S., Mansfeld F., Postyn A., Shih H. (1991). Microbial Ecology of Crown Corrosion in Sewers. J. Environ. Eng..

[59] Herisson J., van Hullebusch E.D., Moletta-Denat M., Taquet P., Chaussadent T. (2013). Toward an accelerated biodeterioration test to understand the behavior of Portland and calcium aluminate cementitious materials in sewer networks. Int. Biodeterior. Biodegrad..

[60] Monteny J., Vincke E., Beeldens A., De Belie N., Taerwe L., Van Gemert D., Verstraete W. (2000). Chemical, microbiological, and in situ test methods for biogenic sulfuric acid corrosion of concrete. Cem. Concr. Res..

[61] Santo Domingo J.W., Revetta R.P., Iker B., Gomez-Alvarez V., Garcia J., Sullivan J., Weast J. (2011). Molecular survey of concrete sewer biofilm microbial communities. Biofouling.

[62] Minz D., Flax J.L., Green S.J., Muyzer G., Cohen Y., Wagner M., Rittmann B.E., Stahl D.A. (1999). Diversity of Sulfate-Reducing Bacteria in Oxic and Anoxic Regions of a Microbial Mat Characterized by Comparative Analysis of Dissimilatory Sulfite Reductase Genes. Appl. Environ. Microbiol..

[63] Ito T., Nielsen J.L., Okabe S., Watanabe Y., Nielsen P.H. (2002). Phylogenetic Identification and Substrate Uptake Patterns of Sulfate-Reducing Bacteria Inhabiting an Oxic-Anoxic Sewer Biofilm Determined by Combining Microautoradiography and Fluorescent In Situ Hybridization. Appl. Environ. Microbiol..

[64] Zhu Z., Chu H., Jiang S., Guo M.-Z., Xu Y., Liang Y., Jiang L. (2022). Improvement in the microbially induced corrosion resistance of concrete sewers using electrodeposition. Cem. Concr. Compos..

[65] Biswal B.K., Wang B., Tang C.-J., Chen G.-H., Wu D. (2020). Elucidating the effect of mixing technologies on dynamics of microbial communities and their correlations with granular sludge properties in a high-rate sulfidogenic anaerobic bioreactor for saline wastewater treatment. Bioresour. Technol..

[66] Vallero M.V.G., Lettinga G., Lens P.N.L. (2005). High rate sulfate reduction in a submerged anaerobic membrane bioreactor (SAMBaR) at high salinity. J. Membr. Sci..

[67] El-Liethy M.A., Hemdan B.A., El-Taweel G.E. (2023). New insights for tracking bacterial community structures in industrial wastewater from textile factories to surface water using phenotypic, 16S rRNA isolates identifications and high-throughput sequencing. Acta Trop..

[68] Wei Y., Li Y., Wang Y., Luo X., Du F., Liu W., Xie L., Chen J., Ren Z., Hou S. (2022). The microbial diversity in industrial effluents makes high-throughput sequencing-based source tracking of the effluents possible. Environ. Res..

[69] Milde K., Sand W., Wolff W., Bock E. (1983). Thiobacilli of the Corroded Concrete Walls of the Hamburg Sewer System. Microbiology.

[70] Kerger B.D., Nichols P.D., Sand W., Bock E., White D.C. (1987). Association of acid-producing thiobacilli with degradation of concrete: Analysis by “signature” fatty acids from the polar lipids and lipopolysaccharide. J. Ind. Microbiol. Biotechnol..

[71] Sand W. (1987). Importance of Hydrogen Sulfide, Thiosulfate, and Methylmercaptan for Growth of Thiobacilli during Simulation of Concrete Corrosion. Appl. Environ. Microbiol..

[72] Munyao O.M., Thiong’o J.K., Muthengia J.W., Mutitu D.K., Mwirichia R., Muriithi G., Marangu J.M. (2020). Study on the effect of Thiobacillus intermedius bacteria on the physico-mechanical properties of mortars of ordinary portland cement. Heliyon.

[73] Sand W., Gehrke T. (2006). Extracellular polymeric substances mediate bioleaching/biocorrosion via interfacial processes involving iron(III) ions and acidophilic bacteria. Res. Microbiol..

[74] Wang Y., Su F., Li P., Wang W., Yang H., Wang L. (2023). Microbiologically induced concrete corrosion in the cracked sewer pipe under sustained load. Constr. Build. Mater..

[75] Huber B., Drewes J.E., Lin K.C., König R., Müller E. (2014). Revealing biogenic sulfuric acid corrosion in sludge digesters: Detection of sulfur-oxidizing bacteria within full-scale digesters. Water Sci. Technol..

[76] Woyciechowski P., Łukowski P., Szmigiera E., Adamczewski G., Chilmon K., Spodzieja S. (2021). Concrete corrosion in a wastewater treatment plant—A comprehensive case study. Constr. Build. Mater..

[77] Parker C. (1945). The corrosion of concrete: 1. The isolation of a species of bacterium associated with the corrosion of concrete exposed to atmospheres containing hydrogen sulphide. Aust. J. Exp. Biol. Med. Sci..

[78] Cho K.-S., Mori T. (1995). A newly isolated fungus participates in the corrosion of concrete sewer pipes. Water Sci. Technol..

[79] Roberts D.J., Nica D., Zuo G., Davis J.L. (2002). Quantifying microbially induced deterioration of concrete: Initial studies. Int. Biodeterior. Biodegrad..

[80] Behnood A., Van Tittelboom K., De Belie N. (2016). Methods for measuring pH in concrete: A review. Constr. Build. Mater..

[81] Schwarz A., Suárez J.I., Aybar M., Nancucheo I., Martínez P., Rittmann B.E. (2020). A membrane-biofilm system for sulfate conversion to elemental sulfur in mining-influenced waters. Sci. Total Environ..

[82] Bock E., Sand W. (1993). The microbiology of masonry biodeterioration. J. Appl. Microbiol..

[83] Wu M., Wang T., Wu K., Kan L. (2020). Microbiologically induced corrosion of concrete in sewer structures: A review of the mechanisms and phenomena. Constr. Build. Mater..

[84] O’Connell M., McNally C., Richardson M.G. (2010). Biochemical attack on concrete in wastewater applications: A state of the art review. Cem. Concr. Compos..

[85] De Windt L., Devillers P. (2010). Modeling the degradation of Portland cement pastes by biogenic organic acids. Cem. Concr. Res..

[86] Okabe S., Odagiri M., Ito T., Satoh H. (2007). Succession of Sulfur-Oxidizing Bacteria in the Microbial Community on Corroding Concrete in Sewer Systems. Appl. Environ. Microbiol..

[87] Gutiérrez-Padilla M.G.D., Bielefeldt A., Ovtchinnikov S., Hernandez M., Silverstein J. (2010). Biogenic sulfuric acid attack on different types of commercially produced concrete sewer pipes. Cem. Concr. Res..

[88] Gu J.-D., Ford T.E., Berke N.S., Mitchell R. (1998). Biodeterioration of concrete by the fungus *Fusarium*. Int. Biodeterior. Biodegrad..

[89] Wilson A.M., Gabriel R., Singer S.W., Schuerg T., Wilken P.M., Van Der Nest M.A., Wingfield M.J., Wingfield B.D. (2021). Doing it alone: Unisexual reproduction in filamentous ascomycete fungi. Fungal Biol. Rev..

[90] Broderick A., Greenshields R. (1981). Sporulation of *Aspergillus niger* and *Aspergillus ochraceus* in Continuous Submerged Liquid Culture. J. Gen. Microbiol..

[91] Colin V.L., Baigorí M.D., Pera L.M. (2013). Tailoring fungal morphology of *Aspergillus niger* MYA 135 by altering the hyphal morphology and the conidia adhesion capacity: Biotechnological applications. AMB Express.

[92] Hayer K., Stratford M., Archer D.B. (2014). Germination of *Aspergillus niger* Conidia Is Triggered by Nitrogen Compounds Related to l-Amino Acids. Appl. Environ. Microbiol..

[93] Jiang L., Pettitt T.R., Buenfeld N., Smith S.R. (2022). A critical review of the physiological, ecological, physical and chemical factors influencing the microbial degradation of concrete by fungi. Build. Environ..

[94] Li K., Li L. (2019). Crack-altered durability properties and performance of structural concretes. Cem. Concr. Res..

[95] Magnuson J.K., Lasure L.L., Tkacz J.S., Lange L. (2004). Organic Acid Production by Filamentous Fungi. Advances in Fungal Biotechnology for Industry, Agriculture, and Medicine.

[96] Geweely N.S.I. (2011). Evaluation of ozone for preventing fungal influenced corrosion of reinforced concrete bridges over the River Nile, Egypt. Biodegradation.

[97] Zhou L., Zhou Y., Hu Y., Cai J., Liu X., Bai C., Tang X., Zhang Y., Jang K.-S., Spencer R.G.M. (2019). Microbial production and consumption of dissolved organic matter in glacial ecosystems on the Tibetan Plateau. Water Res..

[98] George R.P., Ramya S., Ramachandran D., Kamachi Mudali U. (2013). Studies on Biodegradation of normal concrete surfaces by fungus *Fusarium* sp.. Cem. Concr. Res..

[99] Kumar R., Bhattacharjee B. (2003). Porosity, pore size distribution and in situ strength of concrete. Cem. Concr. Res..

[100] Wang Y., Ge Y., Wang X., Chen X., Li Q. (2022). The effect of powder activated carbon on mechanical properties and pore structures of cement-based mortars. Constr. Build. Mater..

[101] Embacher J., Zeilinger S., Kirchmair M., Rodriguez-R L.M., Neuhauser S. (2023). Wood decay fungi and their bacterial interaction partners in the built environment—A systematic review on fungal bacteria interactions in dead wood and timber. Fungal Biol. Rev..

[102] Bhattacharyya S., Akhtar S., Chaudhuri A., Mahanty S., Chaudhuri P., Sudarshan M. (2022). Affirmative nanosilica mediated approach against fungal biodeterioration of concrete materials. Case Stud. Constr. Mater..

[103] Chaudhuri A., Bhattacharyya S., Chaudhuri P., Sudarshan M., Mukherjee S. (2020). In vitro deterioration study of concrete and marble by *Aspergillus tamarii*. J. Build. Eng..

[104] Khan H.A., Castel A., Khan M.S.H. (2020). Corrosion investigation of fly ash based geopolymer mortar in natural sewer environment and sulphuric acid solution. Corros. Sci..

[105] Kong L., Zhao W., Xuan D., Wang X., Liu Y. (2022). Application potential of alkali-activated concrete for antimicrobial induced corrosion: A review. Constr. Build. Mater..

[106] Wei S., Jiang Z., Liu H., Zhou D., Sanchez-Silva M. (2013). Microbiologically induced deterioration of concrete: A review. Braz. J. Microbiol..

[107] Mori T., Nonaka T., Tazaki K., Koga M., Hikosaka Y., Noda S. (1992). Interactions of nutrients, moisture and pH on microbial corrosion of concrete sewer pipes. Water Res..

[108] Gutierrez O., Park D., Sharma K.R., Yuan Z. (2009). Effects of long-term pH elevation on the sulfate-reducing and methanogenic activities of anaerobic sewer biofilms. Water Res..

[109] Joseph A.P., Keller J., Bustamante H., Bond P.L. (2012). Surface neutralization and H_2_S oxidation at early stages of sewer corrosion: Influence of temperature, relative humidity and H_2_S concentration. Water Res..

[110] Wang T., Wu K., Kan L., Wu M. (2020). Current understanding on microbiologically induced corrosion of concrete in sewer structures: A review of the evaluation methods and mitigation measures. Constr. Build. Mater..

[111] Chang H.B., Choi Y.C. (2020). Accelerated performance evaluation of repair mortars for concrete sewer pipes subjected to sulfuric acid attack. J. Mater. Res. Technol..

[112] Madraszewski S., Dehn F., Gerlach J., Stephan D. (2022). Experimentally driven evaluation methods of concrete sewers biodeterioration on laboratory-scale: A critical review. Constr. Build. Mater..

[113] Wells T., Melchers R.E. (2015). Modelling concrete deterioration in sewers using theory and field observations. Cem. Concr. Res..

[114] Huang J., Zhang Y., Sun Y., Ren J., Zhao Z., Zhang J. (2021). Evaluation of pore size distribution and permeability reduction behavior in pervious concrete. Constr. Build. Mater..

[115] Zhang J., Bian F., Zhang Y., Fang Z., Fu C., Guo J. (2018). Effect of pore structures on gas permeability and chloride diffusivity of concrete. Constr. Build. Mater..

[116] Wang Y., Su F., Guo Y., Yang H., Ye Z., Wang L. (2022). Predicting the microbiologically induced concrete corrosion in sewer based on XGBoost algorithm. Case Stud. Constr. Mater..

[117] Huber B., Hilbig H., Drewes J.E., Müller E. (2017). Evaluation of concrete corrosion after short- and long-term exposure to chemically and microbially generated sulfuric acid. Cem. Concr. Res..

[118] Erbektas A.R., Isgor O.B., Weiss W.J. (2019). An accelerated testing protocol for assessing microbially induced concrete deterioration during the bacterial attachment phase. Cem. Concr. Compos..

[119] Raju B., Kumar R., Senthilkumar M., Sulaiman R., Kama N., Dhanalakshmi S. (2022). Humidity sensor based on fibre bragg grating for predicting microbial induced corrosion. Sustain. Energy Technol. Assess..

[120] Li X., Khademi F., Liu Y., Akbari M., Wang C., Bond P.L., Keller J., Jiang G. (2019). Evaluation of data-driven models for predicting the service life of concrete sewer pipes subjected to corrosion. J. Environ. Manag..

[121] Haile T., Nakhla G., Allouche E. (2008). Evaluation of the resistance of mortars coated with silver bearing zeolite to bacterial-induced corrosion. Corros. Sci..

[122] Jin Y., Zhou E., Ueki T., Zhang D., Fan Y., Xu D., Wang F., Lovley D.R. (2023). Accelerated Microbial Corrosion by Magnetite and Electrically Conductive Pili through Direct Fe^0^-to-Microbe Electron Transfer. Angew. Chem. Int. Ed..

[123] Sand W., Bock E. (1984). Concrete corrosion in the Hamburg Sewer system. Environ. Technol. Lett..

[124] Kong L., Liu C., Cao M., Fang J. (2018). Mechanism study of the role of biofilm played in sewage corrosion of mortar. Constr. Build. Mater..

[125] Khan H.A., Khan M.S.H., Castel A., Sunarho J. (2018). Deterioration of alkali-activated mortars exposed to natural aggressive sewer environment. Constr. Build. Mater..

[126] Berndt M.L. (2011). Evaluation of coatings, mortars and mix design for protection of concrete against sulphur oxidising bacteria. Constr. Build. Mater..

[127] Jiang G., Sun X., Keller J., Bond P.L. (2015). Identification of controlling factors for the initiation of corrosion of fresh concrete sewers. Water Res..

[128] Liang Y., Chu H., Guo M.-Z., Zeng Y., Zhu Z., Jiang L. (2021). CTAB-assisted electrodeposition of Cu coating on hardened cement paste for controlling microbial induced concrete corrosion. Constr. Build. Mater..

[129] Karthikeyan C., Varaprasad K., Venugopal S.K., Shakila S., Venkatraman B.R., Sadiku R. (2021). Biocidal (bacterial and cancer cells) activities of chitosan/CuO nanomaterial, synthesized via a green process. Carbohydr. Polym..

[130] Zhu Z., Chu H., Guo M.-Z., Zhang Y., Song Z., Jiang L. (2021). Anti-microbial corrosion performance of concrete treated by Cu_2_O electrodeposition: Influence of different treatment parameters. Cem. Concr. Compos..

[131] Grengg C., Koraimann G., Ukrainczyk N., Rudic O., Luschnig S., Gluth G.J.G., Radtke M., Dietzel M., Mittermayr F. (2021). Cu- and Zn-doped alkali activated mortar—Properties and durability in (bio)chemically aggressive wastewater environments. Cem. Concr. Res..

[132] Kong L., Zhang B., Fang J. (2017). Study on the applicability of bactericides to prevent concrete microbial corrosion. Constr. Build. Mater..

[133] Etim I.-I.N., Dong J., Wei J., Nan C., Daniel E.F., Subedi D.B., Xu D., Yadav A.P., Su M., Ke W. (2020). Mitigation of sulphate-reducing bacteria attack on the corrosion of 20SiMn steel rebar in sulphoaluminate concrete using organic silicon quaternary ammonium salt. Constr. Build. Mater..

[134] Okeniyi J.O. (2016). C_10_H_18_N_2_Na_2_O_10_ inhibition and adsorption mechanism on concrete steel-reinforcement corrosion in corrosive environments. J. Assoc. Arab Univ. Basic Appl. Sci..

[135] Voicu G., Tiuca G.-A., Badanoiu A.-I., Holban A.-M. (2022). Nano and mesoscopic SiO_2_ and ZnO powders to modulate hydration, hardening and antibacterial properties of portland cements. J. Build. Eng..

[136] Yamanaka T., Aso I., Togashi S., Tanigawa M., Shoji K., Watanabe T., Watanabe N., Maki K., Suzuki H. (2002). Corrosion by bacteria of concrete in sewerage systems and inhibitory effects of formates on their growth. Water Res..

[137] Sugio T., Kuwano H., Negishi A., Maeda T., Takeuchi F., Kamimura K. (2001). Mechanism of Growth Inhibition by Tungsten in *Acidithiobacillus ferrooxidans*. Biosci. Biotechnol. Biochem..

[138] Etim I.-I.N., Dong J., Wei J., Nan C., Pokharel D.B., Umoh A.J., Xu D., Su M., Ke W. (2021). Effect of organic silicon quaternary ammonium salts on mitigating corrosion of reinforced steel induced by SRB in mild alkaline simulated concrete pore solution. J. Mater. Sci. Technol..

[139] Alum A., Rashid A., Mobasher B., Abbaszadegan M. (2008). Cement-based biocide coatings for controlling algal growth in water distribution canals. Cem. Concr. Compos..

[140] Juksu K., Zhao J.-L., Liu Y.-S., Yao L., Sarin C., Sreesai S., Klomjek P., Jiang Y.-X., Ying G.-G. (2019). Occurrence, fate and risk assessment of biocides in wastewater treatment plants and aquatic environments in Thailand. Sci. Total Environ..

[141] Arslan H., Aytaç U.S., Bilir T., Şen Ş. (2019). The synthesis of a new chitosan based superplasticizer and investigation of its effects on concrete properties. Constr. Build. Mater..

[142] Guo H., Tian L., Liu S., Wang Y., Hou J., Zhu T., Liu Y. (2023). The potent effects of polyoxometalates (POMs) on controlling sulfide and methane production from sewers. Chem. Eng. J..

[143] Omar O.M., Abd Elhameed G.D., Sherif M.A., Mohamadien H.A. (2012). Influence of limestone waste as partial replacement material for sand and marble powder in concrete properties. HBRC J..

[144] Song Y., Chetty K., Garbe U., Wei J., Bu H., O’moore L., Li X., Yuan Z., McCarthy T., Jiang G. (2021). A novel granular sludge-based and highly corrosion-resistant bio-concrete in sewers. Sci. Total Environ..

[145] Shi H., Liu F., Yang L., Han E. (2008). Characterization of protective performance of epoxy reinforced with nanometer-sized TiO_2_ and SiO_2_. Prog. Org. Coat..

[146] Peyvandi A., Soroushian P., Balachandra A.M., Sobolev K. (2013). Enhancement of the durability characteristics of concrete nanocomposite pipes with modified graphite nanoplatelets. Constr. Build. Mater..

[147] Klapiszewska I., Parus A., Ławniczak Ł., Jesionowski T., Klapiszewski Ł., Ślosarczyk A. (2021). Production of antibacterial cement composites containing ZnO/lignin and ZnO–SiO_2_/lignin hybrid admixtures. Cem. Concr. Compos..

[148] Roy D.M., Arjunan P., Silsbee M.R. (2001). Effect of silica fume, metakaolin, and low-calcium fly ash on chemical resistance of concrete. Cem. Concr. Res..

[149] Usman J., Sam A.M. (2017). Acid resistance of palm oil fuel ash and metakaolin ternary blend cement mortar. Sustain. Environ. Res..

[150] Monteny J., De Belie N., Vincke E., Verstraete W., Taerwe L. (2001). Chemical and microbiological tests to simulate sulfuric acid corrosion of polymer-modified concrete. Cem. Concr. Res..

[151] Janfeshan Araghi H., Nikbin I.M., Rahimi Reskati S., Rahmani E., Allahyari H. (2015). An experimental investigation on the erosion resistance of concrete containing various PET particles percentages against sulfuric acid attack. Constr. Build. Mater..

[152] Do J., Song H., So H., Soh Y. (2005). Antifungal effects of cement mortars with two types of organic antifungal agents. Cem. Concr. Res..

[153] De Muynck W., De Belie N., Verstraete W. (2009). Effectiveness of admixtures, surface treatments and antimicrobial compounds against biogenic sulfuric acid corrosion of concrete. Cem. Concr. Compos..

[154] Maury-Ramirez A., De Muynck W., Stevens R., Demeestere K., De Belie N. (2013). Titanium dioxide based strategies to prevent algal fouling on cementitious materials. Cem. Concr. Compos..

[155] Hayek M., Salgues M., Souche J.-C., Cunge E., Giraudel C., Paireau O. (2021). Influence of the Intrinsic Characteristics of Cementitious Materials on Biofouling in the Marine Environment. Sustainability.

[156] Youssari F.Z., Taleb O., Benosman A.S. (2023). Towards understanding the behavior of fiber-reinforced concrete in aggressive environments: Acid attacks and leaching. Constr. Build. Mater..

[157] Basheer P.A.M., Basheer L., Cleland D.J., Long A.E. (1997). Surface treatments for concrete: Assessment methods and reported performance. Constr. Build. Mater..

[158] Vincke E., Wanseele E.V., Monteny J., Beeldens A., Belie N.D., Taerwe L., Gemert D.V., Verstraete W. (2002). Influence of polymer addition on biogenic sulfuric acid attack of concrete. Int. Biodeterior. Biodegrad..

[159] Kamarul Asri A., Saud S.N., Hamzah E., Ibrahim Z. (2022). In vitro microbiologically-induced concrete corrosion behavior of Ag^+^ loaded zeolite-polyurethane coating for concrete sewer applications. J. Cent. South Univ..

[160] Merachtsaki D., Tsardaka E.-C., Anastasiou E., Zouboulis A. (2021). Anti-corrosion properties of magnesium oxide/magnesium hydroxide coatings for application on concrete surfaces (sewerage network pipes). Constr. Build. Mater..

[161] Haile T., Nakhla G., Allouche E., Vaidya S. (2010). Evaluation of the bactericidal characteristics of nano-copper oxide or functionalized zeolite coating for bio-corrosion control in concrete sewer pipes. Corros. Sci..

[162] Vaidya S., Allouche E.N. (2010). Electrokinetically deposited coating for increasing the service life of partially deteriorated concrete sewers. Constr. Build. Mater..

[163] Roghanian N., Banthia N. (2019). Development of a sustainable coating and repair material to prevent bio-corrosion in concrete sewer and waste-water pipes. Cem. Concr. Compos..

